# Point prevalence of drug prescriptions for elderly and non-elderly inpatients in a teaching hospital

**DOI:** 10.1590/S1516-31802004000200003

**Published:** 2004-03-01

**Authors:** Thais Baleeiro Teixeira Braga, Grace Pfaffenbach, Débora Peterson Leite Weiss, Marilisa Berti de Azevedo Barros, Gun Bergsten-Mendes

**Keywords:** Elderly, Cross-sectional study, Prescription, Inpatients, Pharmacoepidemiology, General hospital, Aging, Idoso, Pacientes internados, Farmacoepidemiologia, Hospitais gerais, Envelhecimento

## Abstract

**CONTEXT:**

Age-related pharmacokinetic and pharmacodynamic changes have been extensively documented, and several concurrent diseases may underlie multiple drug therapy in the elderly. As a result, the risk of adverse drug reactions and drug interactions increases among aged patients. However, only a few studies have compared the prescribing patterns for different age groups of hospitalized patients or have evaluated the effect of age on drug prescription.

**OBJECTIVE:**

To compare the prevalence of drug prescriptions for elderly inpatients, with those for non-elderly inpatients, in order to assess age-related differences in the number of prescribed drugs, drug choices and prescribed doses, and to evaluate the prescription appropriateness for the elderly patients.

**TYPE OF STUDY:**

Cross-sectional survey.

**SETTING:**

400-bed tertiary care general teaching hospital.

**PARTICIPANTS:**

All inpatients on one day of June 1995, except for the Intensive Care Unit and for the Departments of Psychiatry, Pediatrics and Obstetrics and Gynecology.

**PROCEDURES:**

All medicines prescribed to the eligible patients on the study day were recorded from the prescription sheets provided by the hospital pharmacy.

**MAIN MEASUREMENTS:**

Name, therapeutic class, and mean daily dose of the prescribed drugs.

**RESULTS:**

Of the 273 eligible inpatients, 46.5% were 14-44 years old, 33% were 45-64 years old and 20.5% were > 64 years old. Cancer was significantly more frequent among the elderly. The mean number of prescribed drugs was five for all age groups. The five most prescribed drugs for all patients were dipyrone, ranitidine, dipyrone in a fixed- dose combination, metoclopramide and cefazolin. The elderly had significantly more prescriptions for insulin, furosemide and enoxaparin. For most drugs, the mean prescribed dose showed that there was no dose adjustment for elderly patients, and drug choices for this age group were sometimes questionable.

**CONCLUSIONS:**

There was little variation in the prescribing patterns for the elderly when compared with the other age strata.

## RESUMO

**CONTEXTO:**

As alterações farmacocinéticas e farmacodinâmicas associadas ao envelhecimento têm sido amplamente documentadas, bem como o freqüente uso simultâneo de vários medicamentos como conseqüência da elevada prevalência de doenças crônico- degenerativas entre os idosos. Conseqüen-temente, o risco de reações adversas a medicamentos e de interações medicamentosas aumenta em idosos. Entretanto, poucos estudos têm comparado os padrões de prescrição para diferentes grupos etários de pacientes hospitalizados ou avaliaram os efeitos da idade na prescrição de medicamentos.

**OBJETIVO:**

Comparar a prevalência de prescrição de medicamentos para pacientes idosos e não-idosos em um hospital geral universitário a fim de avaliar se existem diferenças relacionadas à idade quanto ao número de medicamentos prescritos, quanto à escolha dos medicamentos e quanto às doses prescritas, bem como a adequação da prescrição para os pacientes idosos.

**TIPO DE ESTUDO:**

Estudo observacional, do tipo transversal.

**LOCAL:**

Hospital-escola, de complexidade terciária, com 400 leitos.

**AMOSTRA:**

Todos os pacientes internados em um dia do mês de junho de 1995, excluindo os internados na Unidade de Terapia Intensiva e dos Departamentos de Psiquiatria, Pediatria, Ginecologia e Obstetrícia.

**PROCEDIMENTO:**

Todos os medicamentos prescritos para os pacientes adultos internados nas enfermarias incluídas no estudo foram registrados a partir da folha de prescrição fornecida pela farmácia hospitalar. Os dados demográficos e os diagnósticos de alta foram fornecidos pelo Departamento de Estatística do Hospital. As informações sobre o peso e exames laboratoriais dos pacientes idosos foram obtidas a partir dos prontuários médicos.

**VARIÁVEIS ESTUDADAS:**

Características demográficas dos pacientes, diagnósticos de alta, exames laboratoriais (somente para os idosos) e medicamentos prescritos (nome, classe terapêutica, dose média diária).

**RESULTADOS:**

Entre os 273 pacientes internados e elegíveis para os estudo, 46,5% encontravam-se na faixa etária de 14-44 anos; 33%, de 45-64 anos e 20,5%, na faixa etária > 64 anos. Neoplasias foram significantemente mais freqüentes entre os pacientes idosos. A média do número de medicamentos prescritos foi de cinco por paciente para os três grupos etários. No conjunto dos pacientes os cinco medicamentos mais prescritos foram: dipirona, ranitidina, dipirona em combinação dose-fixa, metoclopramida e cefazolina. Os pacientes idosos receberam significantemente mais prescrições de insulina, furosemida e enoxaparina. Para a maioria dos medicamentos, a dose média prescrita mostrou que não houve ajuste de dose para os pacientes idosos. Em alguns casos, a escolha de medicamentos para os pacientes idosos foi questionável.

**CONCLUSÕES:**

Este estudo transversal avalia de modo simples e pouco dispendioso a exposição a medicamentos em ambiente hospitalar. Embora o processo de envelhecimento modifique a farmacologia de muitos fármacos, não foram observadas alterações nos padrões de prescrição em função da idade.

## INTRODUCTION

Brazil is getting older. In 1980 the population over 64 years old numbered 4.8 million people (4.0% of the population), which had risen to 8.9 million by 2001 (5.3% of the population).^1^ This increase in the elderly population has lead to more hospital admissions, longer hospital stays, more need for ambulatory and home care and more extensive drug therapies.^2^ For example, from January through November 2001, although the elderly represented 5.3% of the population, this group accounted for 14.4% of all hospital admissions and 18.2% of the hospitalization costs.^1^

The impairing of physiological homeostatic mechanisms, along with progressive pharmacokinetic and pharmacodynamic changes in the aging organism, has been extensively documented over the course of the aging process. Consequently, several concurrent diseases may underlie multiple drug therapy.^3,4^ As a result, the risk of adverse drug reactions and drug interactions increases among the elderly.^5^

Information on drug prescription patterns for elderly inpatients or the effect of age on quantitative and qualitative aspects of drug prescription in hospital settings is scarce.^6,7^ This study assessed whether there are age-related differences in prescribing patterns for hospitalized patients in a general teaching hospital.

## METHODS

A cross-sectional study covering a 24-hour period was performed in a 400-bed tertiary care teaching hospital in June 1995, in order to determine the point prevalence of drug prescription for all inpatients, except from the intensive care unit and psychiatric, pediatric, obstetric and gynecological wards. Patients were classified into three age groups: 14-44 years old (young), 45-64 years old (middle age) and > 64 years old (elderly). The main discharge diagnoses were coded according to the International Classification of Diseases (9^th^ revision).^8^ Weight, plasma creatinine and plasma albumin levels of the elderly patients were recorded from their medical charts. Creatinine clearance was estimated using the formula in Cockcroft & Gault.^9^ The drug prescriptions recorded on the prescription sheets provided by the hospital pharmacy were noted on summary forms. The drugs were classified according to the Anatomical Therapeutic Chemical Classification Index recommended by the World Health Organization Drug Utilization Research Group.^10^

The point prevalence of prescription was determined by using the following formula:


$$point\;prevalence=\frac{\begin{array}{c} \text{number of patients with a prescription}\\ \text{for the drug on the study day} \end{array}}{\text{total number of patients}}$$

The mean daily doses of the most prescribed drugs were determined for each age group. For the evaluation of drug prescription appropriateness for elderly patients, the number of prescribed drugs, the drug choice among therapeutic alternatives and the mean prescribed dose were considered.

Statistical analysis was performed to detect differences among age-strata using the chisquared test and the Fisher exact test. Analysis of variance (ANOVA) was used for detecting the variation among the means of prescribed drugs in the three age-strata. When applicable, the Duncan test was used for expressing the contrast between means. The statistical differences were considered significant when p ≤ 0.05.

## RESULTS

### The patients and their morbidity patterns

Of the 273 eligible patients, 127 (46.5%) belonged to the young age group, 90 (33%) to the middle age group, and 56 (20.5%) were elderly patients. One hundred and seventy-five patients (64.1%) were male. The weights of 34 elderly patients (60.7%) had already been recorded on the medical charts, and the mean weights for men and women were 67 ± 10 kg and 65 ± 14 kg, respectively. Nine per cent of the patients weighed less than 55 kg. The plasma creatinine levels of 44 elderly patients (78.6%) had been recorded on the medical charts and were above the reference values in 24 patients. The estimated creatinine clearance of 38% of the elderly men and 18% of the elderly women was ≤ 39 ml/min/1.73 m^2^. The plasma albumin levels had been recorded for 8 elderly patients (14.3%), and in 5 (62.5%) the levels were < 3.4 g/dl.

The five most frequent discharge diagnoses were: 1) for the young age group: digestive diseases (18.9%), cancer (12.6%), respiratory diseases (10.2%), injury and poisoning (10.2%) and infectious diseases (9.5%); 2) for the middle age group: cardiovascular diseases (31.1%), cancer (22.2%), genitourinary diseases (8.9%), injury and poisoning (6.7%) and central nervous system diseases (6.7%); and 3) for the elderly: cancer (26.8%), cardiovascular diseases (23.2%), digestive diseases (10.7%), injury and poisoning (8.9%) and genitourinary diseases (8.9%). The elderly had significantly more cancer (26.8%; p = 0.04); the middle age group significantly more cardiovascular diseases (31.1%; p < 0.01); and the young age group significantly more digestive system diseases (18.9%; p = 0.002) and infectious diseases (9.5%; p = 0.03), when compared with the other groups. There were no age differences for the other diagnoses.

### Prescription patterns and age

**Figure 1:**
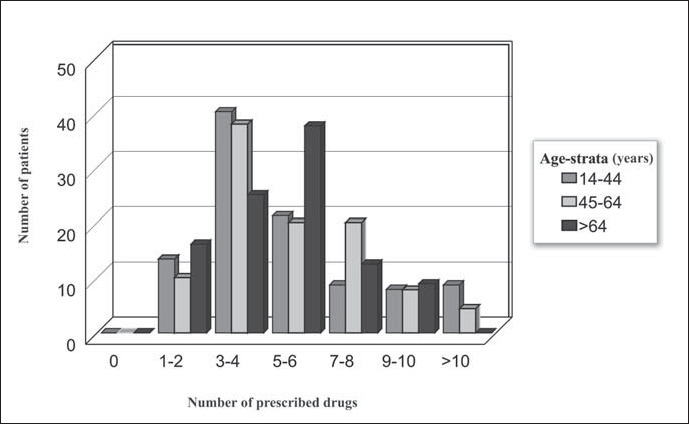
Distribution of the number of prescribed drugs according to age group (years).

A mean of five prescribed drugs was found for all age groups. The median number of prescribed drugs was 4 (range 1-21) for the young age group, 5 (range 1-13) for the middle age group, and 5 (range 1-10) for the elderly patients. Among the elderly patients, 58.9% received five drugs or more, compared with 52.2% for the middle age group and 46.5% for the young age group (Figure 1). These differences were not significant.

The seven most prescribed therapeutic classes were (overall prevalence; prevalence among the elderly): N — central nervous system (81.3%; 73.2%), A — alimentary tract and metabolism (72.9%; 69.6%), J — antiinfectives for systemic use (52.4%; 48.2%), C — cardiovascular system (41.0%; 62.5%), B — blood and blood forming organs (25.3%; 41.1%), R — respiratory system (15.0%; 17.9%) and P — antiparasitics (17.9%; 19.6%). For classes N, A, J and R, there were no significant age-related differences in the number of prescriptions. The elderly had significantly more prescriptions for classes B and C. The 13 most prescribed drugs corresponding to the seven most prescribed therapeutic classes are given in Table 1. The most prevalent drug prescription among the three age groups was for dipyrone followed by ranitidine. The prevalence of prescriptions for insulin, furosemide and enoxaparin was significantly higher for the elderly age group.

**Table 1:** Prevalence (%) among the age-strata and the overall prevalence (%) of the most prescribed drugs within the most prescribed therapeutic classes

Therapeutic classes*	Drug	Prevalence				
		14 - 44	45 - 64	> 64	overall	p value†
**N**	Dipyrone	54.3	48.9	35.7	48.7	NS
	Dipyrone, combination‡	30.7	33.3	32.1	31.9	NS
	Paracetamol, combination§	13.4	13.3	16.1	13.9	NS
	Diazepam	7.1	6.7	12.5	8.1	NS
**A**	Ranitidine	44.1	50.0	42.9	45.8	NS
	Metoclopramide	38.6	26.7	25.0	31.9	NS
	Insulin	3.2	12.2	21.4	9.9	< 0.001
**J**	Cefazolin	22.0	15.6	21.4	19.8	NS
**C**	Nifedipine	11.0	21.1	19.6	16.1	NS
	Captopril	3.9	17.8	14.3	10.6	NS
	Furosemide	7.1	18.9	23.2	14.3	0.005
**B**	Enoxaparin	0.8	6.7	23.2	7.3	< 0.001
**R**	Aminophylline	3.9	10.0	10.7	7.3	NS
**P**	Metronidazole	11.0	7.8	10.7	9.9	NS

* *According to the Anatomical Therapeutic Chemical Classification Index of the World Health Organization*^9^;

† *The p values correspond to comparisons among age-strata (chi-squared test); NS = not significant;*

‡ *Dipyrone, combination: (1 ampoule) = 500 mg dipyrone + 25 mg promethazine + 25 mg adiphenine;*

§ *Paracetamol, combination: (1 tablet) = 500 mg paracetamol + 7.5 mg or 30 mg codeine*

The mean doses for almost all of the most prescribed drugs for the three age groups were not significantly different, with a few exceptions (Table 2). The mean prescribed dose of parenteral metoclopramide for the elderly was 33.8 ± 5.1 mg (range 30-40), which was significantly higher than for other age strata (p = 0.03; ANOVA, Duncan test). The mean prescribed dose of nifedipine for the young age group was 72.0 ± 11 mg (range 60-80), for the middle age group, 54.3 ± 22.3 mg (20-80), and for the old age group, 48.3 ± 18.4 mg (range 30-80). The dose of nifedipine was significantly higher among the young age group (p = 0.005; ANOVA, Duncan test). About 60% of the prescriptions for nifedipine were on a *prn* (*pro re nata*) basis for acute elevations of blood pressure. There was a tendency to prescribe diazepam for the elderly more frequently (12.5% against 7.1% and 6.7%), and at a lower dose, although the differences were not significant. Similarly, the doses of aminophylline tended to be reduced for the elderly, but again the differences were not significant.

**Table 2:** Mean prescribed dose for the most prescribed drugs according to age strata

Drug	Route	14 – 44 (years)		45 – 64 (years)		> 64 (years)	
		Mean dose ± SD	range	Mean dose ± SD	range	Mean dose ± SD	range
Dipyrone	iv	3.85 ± 0.53 g	1.0 - 4.0	4.0 g	4.0	3.33 ± 0.98 g	2.0 - 4.0
Ranitidine	oral	300 ± 96.5 mg	150 - 600	288 ± 75.5 mg	150 – 600	283 ± 90.1 mg	150 – 450
Metoclopramide*	iv	30.5 ± 4.3 mg	10 - 40	31.0 ± 3.0 mg	30 – 40	33.8 ± 5.1 mg	30 – 40
Dipyrone, comb. †	iv	3.0 ± 0.4 amp	1 - 4	3.0 ± 0.3 amp	2 – 4	3.0 ± 0.3 amp	2 - 4
Cefazolin	iv	3.0 g	3.0	3.0 g	3.0	2.8 g ± 0.6 g	1 - 3
Paracetamol, comb. ‡	oral	3.7 ± 1 tab	2 - 6	3.3 ± 1 tab	1 – 4	3.1 ± 1 tablet	1 - 4
Nifedipine**	oral	72 ± 11 mg	60 - 80	54.3 ± 22.3 mg	20 – 80	48.3 ± 18.4 mg	30 - 80
Metronidazole	iv	1.5 ± 0.1 g	1 - 1.5	1.5 g	1.5	1.5 g	1.5
Furosemide	oral	53.3 ± 23.1 mg	40 - 80	69.1 ± 36.2 mg	40 – 120	50 ± 20 mg	40 - 80
Diazepam	oral	15 ± 11 mg	10 - 40	10.6 ± 7.1 mg	2.5 – 20	8.3 ± 2.6 mg	5 - 10
Captopril	oral	43 ± 31 mg	12.5 - 100	36 ± 33 mg	12.5 –150	38 ± 30 mg	12.5 - 100
Aminophylline	iv	690 ± 60 mg	600 - 720	680 ± 69 mg	600 – 720	660 ± 120 mg	480 - 720
Regular insulin	iv	31.25 ± 19.31 U	5 – 50	10.60 ± 10.49 U	3 – 40	11.70 ± 5.87 U	5 - 20
Enoxaparin	iv	20 mg	20	20 mg	20	21.5 ± 5.5 mg	20 - 40

† *1 ampoule = 2 ml = 750 mg dipyrone + 25 mg adiphenine + 25 mg promethazine.*

‡ *1 tablet = 500 mg paracetamol + 30 mg codeine; iv = intravenous; amp = ampoule. The p values correspond to significant differences among the 14-44 and 45-64 age-strata in comparison with the over-64 age stratum (ANOVA and Duncan test):*

* *p= 0.03;*

** *p = 0.005*

## DISCUSSION

This study used the point prevalence to evaluate drug exposure in a hospital setting and showed that relevant information can be obtained with a one-day assessment of the drug prescription pattern. It is an easy and inexpensive method that does not require online prescriptions or well-structured databases.

Few studies have evaluated the influence of age on hospital-based drug exposure. Some studies have shown that elderly inpatients receive more drugs than younger ones.^5-7,11^ while other studies have found little variation in the prescribing patterns for the elderly.^12,13^ Different studies have used different methods and their results are difficult to compare. Using a cross sectional design, Christopher et al. (1978) reported an average of 3.3 drugs per elderly patient in Dundee hospitals.^11^ Although the mean number of prescribed drugs was not significantly different among the three age groups in our study, there was a tendency for elderly patients to receive more drugs. However, considering the old people’s morbidity, a mean of five drugs cannot be said to be inappropriate. Similarly, the mean of five drugs (range of 1-21) prescribed for the young age group probably reflected the complexity of the health care provided by this hospital.

Only two fixed-dose combinations were found. A prescription for a fixed-dose combination of dipyrone, promethazine and adiphenine as shown in Table 1, was given to 31.9% of the patients, as an analgesic. This combination lacks pharmacological rational- ity,^14,15^ since promethazine does not have analgesic properties by itself, nor does it enhance the analgesic effect of dipyrone. On the other hand, it exposes the patients, and especially the elderly, to significant adverse drug reactions as a consequence of the antimuscarinic and sedative properties of promethazine.^15-17^ The fixed- dose combination of paracetamol with codeine that was given to 13.9% of the patients has the disadvantage of not allowing for the necessary dose adjustment for frail old patients.^18^

When analyzing the appropriateness of drug prescriptions for elderly patients, two aspects of paramount importance are dose adjustment and drug selection.

### Dose adjustment

- Aminophylline: the dose should be adjusted according to the patient’s weight in order to avoid adverse drug reactions;^16^ the maximum dose of aminophylline for the elderly should be 500 mg/day, less than the value found in this study (660 ± 120 mg).
- Metoclopramide: the recommended daily dose for the elderly is up to 15 mg/day,^19^ i.e. half the dose found in this study (33.8 ± 5.1 mg); doses greater than this recommended dose expose the elderly to the risk of confusion and extrapyramidal effects.^19-22^
- Nifedipine: the mean dose prescribed to the elderly (48.3 ± 18.4 mg) was above the maximum recommended dose of 40 mg/day.^19^
- Cefazolin: no age-related dose adjustment was observed^23^ even though 46.8% of the elderly had an estimated creatinine clearance < 50 ml/min/1.73 m^2^.^24^
- Metronidazole: although the dose should be reduced in cases of renal or hepatic dysfunction, the mean prescribed dose was the same for all patients.^23,25^
- Ranitidine: the dose for the elderly should be half the dose prescribed for non-eld- erly; the mean dose was the same for all patients, irrespective of age.^23^

### Drug selection

- Ranitidine: this study did not address the reason for the high prevalence of ranitidine prescription (42.9%), but it is noteworthy that only 10.7% had digestive diseases.
- Nifedipine: the use of this short-acting calcium channel blocker for the treatment of hypertension in the aged should be reevaluated, since the elderly treated with nifedipine are at greater risk of dying from cardiovascular diseases,^24,26-28^ probably as a result of excessive vasodilatation that can lead to severe hypotension.
- Diazepam should not be prescribed for elderly patients, since this long-acting benzodiazepine drug exposes them to the risk of falls and hip fracture.^29^
- Enoxaparin: the advantages of this low- molecular-weight heparin over conventional treatment with heparin are questionable,^30,31^ especially when considering that enoxaparin is so many times more expensive than heparin. The safety of enoxaparin has not been established for this age group.^31^

## CONCLUSION

This cross-sectional study showed that certain aspects of drug prescribing, especially for the elderly, need to be modified in order to provide more rational therapy. The results from this study indicate that in any setting of medical care, the elderly need to be treated individually, particularly with regard to pharmacokinetics and pharmacodynamics. Guidelines for the geriatric prescription of many drugs have already been established. Rational individualized drug prescription is a challenge that poses the need to take into account age-related modifications in physiological characteristics and the occurrence of concomitant diseases. A continuing medical program in therapeutics, designed to expose prescribers to up-to-date and clinically relevant information on drug therapy, could help in reaching the goal of safe and effective pharmacotherapy for the elderly.
